# Outpatient coordination reform improves the sustainability of China's Urban Employee Basic Medical Insurance Fund

**DOI:** 10.3389/fpubh.2024.1446408

**Published:** 2024-11-26

**Authors:** Jingping Lin, Chengming Yan, Hanning Wang, Cong Xu, Weiqiang Fan

**Affiliations:** School of Economics and Management, East China Jiaotong University, Nanchang, China

**Keywords:** basic medical insurance for urban employees, outpatient co-ordination, fund sustainability, Employee Medical Insurance Pooling Fund Model, fund balance

## Abstract

This study aims to examine the impact of outpatient coordination reform (OCR) on the sustainability of the Urban Employee Basic Medical Insurance (UEBMI) pooling fund. Using an employee medical insurance pooling fund model, we found that, without OCR, the fund will face current deficits by 2024 and cumulative deficits by 2033. However, with the “partial outpatient coordination reform,” offering 50 or 75.6% reimbursement rates, the onset of cumulative deficits is delayed by 11 and 8 years, respectively. In contrast, implementing the “complete outpatient coordination reform” with 50 or 75.6% reimbursement rates ensures a positive cumulative balance until 2050. In conclusion, OCR not only strengthens the mutual assistance function of medical insurance but also ensures the sustainability of the insurance fund. Therefore, it is advisable to implement and refine these policies promptly to reduce the financial burden on the UEBMI pooling fund.

## 1 Introduction

In 1998, China witnessed the formal establishment of the Urban Employees Basic Medical Insurance Fund (UEBMIF) system, replacing the employer-based social security system that had been in place for nearly half a century and transitioning to a model combining pooling funds and individual funds. Employers primarily contribute to the pooling fund and operate on a pay-as-you-go basis. It is mainly used to reimburse medical expenses urban employees incur for major illnesses during hospitalization and outpatient care. Its key function is social risk-sharing, facilitated by the pooling fund, which mitigates the financial risk of individual major medical expenses through collective funding. In contrast, individual funds operate on a fully accumulated basis, which means that the funds belong to the individual employee and are mainly used for expenses at designated medical institutions, outpatient medical costs, and purchasing drugs at designated pharmacies. Additionally, these funds can be rolled over and inherited. In comparison to the limited and personalized nature of individual funds, the pooling funds play a crucial role in ensuring the stability and sustainability of the overall healthcare system, serving as a fundamental pillar of the medical insurance fund. With the continuous deepening of socioeconomic development, the current society is presenting new trends, such as population aging, expansion of outpatient medical services, rapid development of medical technology, and chronicization of diseases. At the same time, the shift in the new health concept of “prevention is better than cure” has led to a rapid increase in outpatient demand, resulting in a sharp rise in employee outpatient expenses. The latest data shows that the average outpatient expenses per visit in 2022 were more than double those in 2010. From a practical perspective, insured urban employees face a dilemma where the sick have inadequate coverage, while the healthy cannot access their benefits. The data from the 2022 National Development Statistics Bulletin of Medical Security show that the accumulated balance of employee individual fund accounts constitutes 38.7% of the total medical insurance pooling fund for employees. Conversely, employees who suffer from serious illnesses often find their health accounts insufficiently funded to cover their medical expenses. This has resulted in the problem of inefficient allocation of the medical insurance pooling fund. On one hand, the previous system, in which the medical insurance fund was allocated to individual fund accounts for the sole use of the insured, is struggling to meet the current outpatient medical needs. On the other hand, for urban employees, the system fails to fulfill its role in mutual aid and risk-sharing, resulting in the waste of resources. Therefore, it is imperative to reform individual fund accounts to alleviate the burden of outpatient medical expenses on urban-insured employees, especially older adults. In 2020, the “Opinions of the Central Committee of the Communist Party of China (CPC) Central Committee and the State Council on Deepening the Reform of the Medical Security System” proposed gradually incorporating outpatient medical expenses into the payment scope of the UEBMI pooling fund, reforming the individual fund of the UEBMI, and establishing a sound outpatient coordination security mechanism. In 2021, the “Guiding Opinions of the General Office of the State Council on Establishing and Improving the Mechanism for Outpatient Coordination under Employee Basic Medical Insurance” clearly proposed to establish and improve the UEBMI outpatient expense pooling mechanism. Under this reform, the premiums paid by employers were to be fully allocated to the pooling fund, with the increased portion of the pooling fund primarily used for outpatient coordination security. The following two reform schemes are defined to provide a more intuitive comparison of the impact of OCR policies on the sustainability of the UEBMI pooling fund: The first is termed “Partial Outpatient Coordination Reform,” which aligns with the current policies in China. Under this scheme, the employer's contributions to the UEBMI are fully allocated to the pooling fund, while individual contributions remain in individual funds. This scheme is currently being implemented in the majority of provinces across China. The second is called “Complete Outpatient Coordination Reform.” This radical scheme involves abolishing personal funds and transferring both employer's and employee's contributions to the pooling fund. Currently, after a transition period of approximately 3 years, the majority of the provinces have gradually introduced OCR based on their own actual situations. Therefore, what kind of impact will the implementation of OCR have on the sustainability of the medical insurance pooling fund for employees? Will it improve or worsen it? To answer this inquiry, this study focuses on the UEBMI pooling fund. It analyzes the impact of OCR implementation before 2050 on fund sustainability by establishing an Employee Medical Insurance Pooling Fund Model. The remaining sections of this article are arranged as follows: Section 2 will cover a literature review. Section 3 will introduce the construction and parameter setting of the Employee Medical Insurance Pooling Fund Model. Section 4 will elucidate the simulation results and analysis of the adjustment of OCR policies on the medical insurance fund for an urban employee. Section 5 will present the conclusion and policy implications.

## 2 Literature review

Basic medical insurance for urban employees, as an essential part of the social security system, is closely related to the degree of population aging. With the increasing aging population, the sustainability of the medical insurance pooling fund for urban employees will inevitably be impacted. Currently, the academic research on the sustainability of the UEBMIF mainly focuses on three main aspects: population aging, fertility policies, and delayed retirement. Regarding the impact of population aging, the majority of scholars have found that continuous aging of the population will jeopardize the sustainable operation of the medical insurance pooling fund (1–3). Based on empirical analysis, Yu Bin (4) concluded that aging will render the UEBMI pooling fund unsustainable by 2060, gradually losing their ability to operate independently. Regarding adjustments in fertility policies, the majority of scholars believe that increasing fertility rates will affect the sustainability of funds. Jun et al. (5) and Yi et al. (6) used actuarial models to conclude that adjustments in fertility policies can reduce the cumulative deficit size of the UEBMI pooling fund. The research results of Yuantao et al. (18) indicate that the “universal two-child” policy can potentially keep the balance of the medical insurance fund for urban employees positive. However, the study by Changchun et al. (7) indicates that the specific effects of fertility policies on the medical insurance fund depend on the contribution rate. Regarding implementing delayed retirement policies, delaying retirement can alleviate the pressure on medical insurance fund expenditures in the short term. Nevertheless, in the long term, delayed retirement policies will not change the ultimate deficit situation of the funds. Shu (8) concluded that delaying retirement by 6 months can delay the time when the fund goes into deficit by 2 years and reduce the cumulative deficit by 31.63% based on the actuarial models of the UEBMI pooling fund. Li and Jing (9) concluded that delayed retirement policies can postpone the occurrence of deficits in the funds by 3 years using dynamic actuarial models. Jin and Zhen (10) and Chao (11) found that delaying retirement can only alleviate the expenditure pressure on the medical insurance pooling fund in the short term and cannot fundamentally solve the problem of the deficit, making it necessary to reform the medical insurance system for the long-term development of medical insurance fund. In addition, to improve the sustainability of the UEBMI pooling fund, Yating and Zhe (17) suggested reforming the medical insurance individual fund for employees and implementing outpatient coordination mechanisms.

In summary, scholars have made fruitful achievements in the research on the sustainability of the UEBMI pooling fund, and they have also put forward policy measures to address its future deficits. Nevertheless, two areas remain underexplored. First, while the majority of the existing studies focus on the sustainability of the UEBMI pooling fund—primarily from the viewpoints of population aging, fertility policies, and delayed retirement—there is a notable lack of studies that examine the issue from the perspective of outpatient coordination reform. Second, there is also a deficiency in research into the impact of different outpatient expense reimbursement ratios on the sustainability of the medical insurance pooling fund. Based on the aforementioned expansion space, the innovations of this study mainly include the following two points: first, based on the UEBMI pooling fund models, the number of insured urban employees is predicted, and the sustainability of the UEBMI pooling fund under different outpatient coordination reform schemes before 2050 is analyzed, providing theoretical references for the implementation of the future OCR policies. Second, considering different outpatient expense reimbursement ratios, this study analyzes the sustainability of the funds, providing references for exploring ways to improve the sustainability of the funds in the future.

## 3 Model construction and parameter assumptions

The Urban Employee Basic Medical Insurance Fund consists of two parts, namely, the pooling fund and the individual fund. According to the “Guiding Opinions of the State Council General Office on Establishing and Improving the Mechanism of Outpatient Mutual Assistance for Basic Medical Insurance for Urban Employee” (State Office [2021]No. 14), the basic medical insurance premiums paid by employers are all included in the pooling fund. According to Chinese policy, the insured person is required to cover the medical expenses out of pocket when an employee's individual funds are insufficient. Therefore, individual funds will remain balanced. For this reason, this study primarily explores the sustainability of the pooling fund. After adjusting the structure of the pooling fund and individual fund, the increased pooling fund is mainly used for outpatient mutual assistance to improve the outpatient treatment of insured persons. Before establishing the UEBMI pooling fund model, according to the retirement age requirements of different categories, urban employees are divided into three categories: male, female cadres, and female workers, represented by *j* = 1, 2, and 3, respectively. The differences in retirement ages between male and female cadres and female workers are primarily due to historical reasons and varying social roles in China.

### 3.1 Employee Medical Insurance Pooling Fund Model

#### 3.1.1 Pooling fund revenue mode

The revenue of urban employees' basic medical insurance pooling fund in the year *t* is equal to the total number of insured employees in the year *t* multiplied by the contribution base in the year *t* multiplied by the medical insurance contribution rate in the year *t* and multiplied by the proportion of medical insurance fund transferred into the pooling fund account in the year of *t*; the formula is as follows:


$$\begin{array}{l} AI_{t}=\overset{3}{\underset{j=1}{\sum }}\overset{b_{j}-1}{\underset{x=a}{\sum }}N_{t,x}^{j}\times w_{t_{0}-1}\times \overset{t}{\underset{s=t_{0}}{\prod }}\left(1+k_{s}^{1}\right)\times P_{t}^{1}\times P_{t}^{2} \end{array}$$

where *AI*_*t*_ represents the pooling fund revenue for the year *t*; *j* = 1, 2, and 3 denotes male cadres, female cadres, and female workers, respectively; *a* serves as a representative of the starting working age when the workers begin to cover the medical insurance; *b*_*j*_ represents the retirement age of employees who belong to the category of *j*; $N_{t,x}^{j}$ represents the number of *x*-year-old urban employees who fit into the category of *j* for the year *t*; *w*_*t*_0_−1_ denotes the base period wage contribution base for insured employees; $P_{t}^{1}$ refers to the medical insurance contribution rate; $P_{t}^{2}$ refers to the proportion of the pooling fund in the UEBMI; $k_{s}^{1}$ represents the growth rate of the annual medical insurance contribution base for the year, *s*; and *t*_0_ is the start time of the medical insurance fund.

#### 3.1.2 Pooling fund expenditure model

The pooling fund expenditure of UEBMI is equal to the sum of hospitalization expenses reimbursement, maternity fund expenses, and outpatient expenses reimbursement; the formula is as follows:


$$\begin{array}{l} AC_{t}=\sum_{j=1}^{3}\sum_{x=a}^{c}N_{t,x}^{j}\times m_{t_{0}-1}\times \prod_{s=t_{0}}^{t}\left(1+k_{s}^{2}\right)\times u_{t}+Q_{t-1}\\ \times \left(1+\alpha \right)+\sum_{j=1}^{3}\sum_{x=a}^{c}N_{t,x}^{j}\times s_{t_{0}-1}\times \prod_{s=t_{0}}^{t}\left(1+l_{s}^{2}\right)\times r_{t} \end{array}$$

where *AC*_*t*_ represents pooling fund expenditure of UEBMI for the year *i* with *c* as the longest survival age of workers participating in the medical insurance; $\overset{3}{\underset{j=1}{\sum }}\overset{c}{\underset{x=a}{\sum }}N_{t,x}^{j}$ denotes the total number of all insured workers for the year *t* (including retired and active employees); *m*_*t*_0_−1_ represents the average hospitalization expenses of urban employees in the base period; *u*_*t*_ represents the reimbursement rate of the medical insurance for urban employees; $k_{s}^{2}$ is the growth rate of urban employees; *Q*_*t*−1_ represents maternity insurance fund expenditure in the year of *t*−1; α refers to the growth rate of maternity insurance fund expenditure; *s*_*t*_0_−1_ indicates the per capita outpatient expenses of urban employees in the base period; $l_{s}^{2}$ denotes the growth rate of per capita outpatient expenses of urban employees; and *r*_*t*_ refers to the outpatient expense reimbursement rate. If the outpatient coordination reform policy is not implemented, then *r*_*t*_=*0*.

#### 3.1.3 Pooling fund cumulative surplus model

The accumulated balance of the pooling fund of UEBMI in the year *t* is equal to the sum of the accumulated balance of the pooling fund in the previous year (including interest) and the current balance of the pooling fund in the year *t*; the formula is as follows:


$$\begin{array}{l} F_{t}=F_{t-1}\times \left(1+i_{1}\right)+\left[AI_{t}-AC_{t}\right]\times \left(1+i_{2}\right) \end{array}$$

where *F*_*t*_ indicates the accumulated balance of the pooling fund of UEBMI in the year *t*; *i*_1_ represents the 3-month fixed deposit rate of banks in China; and *i*_2_ denotes the interest rate of bank demand deposits.

### 3.2 Parameter assumptions

#### 3.2.1 Number of insured employees

This study uses data from the Seventh National Population Census 2020 to predict the future total population through the cohort component method. The population prediction involves the following three steps: First, multiply the population numbers from the previous year, categorized by age, gender, and area (rural or urban), by the corresponding survival rates to calculate the current natural population increase across these demographic segments. Second, the average number of childbearing-age women categorized by age and area (rural or urban) in the previous year is multiplied by the corresponding fertility rate to access the current number of newborns grouped by age and area (rural or urban). Third, by considering population migration factors and assuming an urbanization rate of 1%, the number of permanent residents is calculated by age, gender, and area (rural or urban).

Based on the predicted total population, predicting the number of urban employees participating in the UEBMI follows three steps: First, it is assumed that the age distribution of active employees participating in the UEBMI in 2022 is consistent with the age distribution of urban residents aged 20–59 in 2022 to obtain the number of active employees participating in the UEBMI by age and gender. Second, assuming that the age distribution of retired employees participating in the UEBMI in 2022 is consistent with urban residents aged 50–100 years in the same year, the number of retired employees participating in the UEBMI can be determined by age and gender. Third, The current number of employees by age and gender is obtained by multiplying the number of employees from the previous year, categorized by age and gender, by the corresponding survival rates while also accounting for the number of new participants joining the UEBMI each year.

#### 3.2.2 Age parameters

The main participants in the UEBMI are urban employees, among whom the proportion of university graduates can reach 90% (12). Although the *Labor Law of the People's Republic of China* stipulates that the minimum employment age is 16 years, the actual employment age of residents has been delayed due to the extension of education years. According to the study by Weiqiang and Hualei (13), the age at which urban employees start working is set at 20 years old. Based on the data from the Seventh *National Population Census* of 2020 and the *China Population Statistical Yearbook* of previous years, the urban employees' maximum survival age is 100 years. According to the *Interim Measures of the State Council on the Placement of Old, Weak, Sick, and Disabled Cadres* and the *Interim Measures of the State Council on the Retirement and Resignation of Workers* (State Council [1978] No. 104), the retirement age of urban employees is set at 60 years for men, 55 years for female cadres, and 50 years for female workers.

#### 3.2.3 Payment base and contribution rate

According to the *Decision of the State Council on Establishing the System of Basic Medical Insurance for Urban Employee* (State Council [1998] No. 44), the basic medical insurance premiums are jointly paid by employers and employees, with an employer contribution rate of 6% and an employee contribution rate of 2%. The contribution base of the basic medical insurance for urban employees is the average wage of the on-the-job workers in the previous year. At the same time, referring to the research of Chao (11) and Hualei et al. (14), the growth rate of the contribution base of the basic medical insurance for urban employees is equal to the growth rate of GDP, which means that the actual contribution base growth rate is 6.5% from 2024 to 2025, and then decreases by 0.5% every 5 years until it reaches 4%.

#### 3.2.4 Hospitalization and outpatient expenses

According to the *2022 National Medical Security Development Statistical Bulletin*, the average hospitalization expense per employee in 2022 was 2,374.82 yuan. According to the study by Hui et al. (15), the average outpatient expense under the implementation of OCR is 585.63 yuan. Therefore, this study assumes 585.63 yuan as the average outpatient expense for 2023. According to the study by Wenjiong et al. (16), the average wage growth rate is generally 1% lower than the growth rate of average hospitalization expenses per capita. Therefore, this study assumes that the growth rate of average hospitalization expenses per capita and the growth rate of average outpatient expenses per capita are 1% higher than the average wage growth rate.

#### 3.2.5 Reimbursement ratio

Since the proportion of actual hospitalization expenses paid by the fund was not announced in 2022, this study assumes, based on the research of scholars like Hualei et al. (14), that the actual reimbursement ratio for hospitalization expenses in 2022 is the same as that of 2019, which is 75.6%, and remains unchanged thereafter.

#### 3.2.6 Maternity insurance

Expenses before and after childbirth for childbearing-age women can be reimbursed through maternity insurance. According to the *Opinions of the State Council General Office on the Comprehensive Promotion of the Merger and Implementation of Maternity Insurance and Employee Basic Medical Insurance* (State Office [2019] No. 10), maternity insurance and employee basic medical insurance were merged and implemented in 2019. Therefore, referring to the expenditure data of maternity insurance funds from the *National Medical Security Development Statistical Bulletin* from 2019 to 2022, this study assumes that the average annual growth rate of expenditure of maternity insurance funds in the future years is 6.3%.

#### 3.2.7 Bank rate

According to data released by the People's Bank of China, the 3-month fixed deposit rate of banks is set at 1.15%, and the interest rate for demand deposits in banks is 0.2%.

## 4 Simulation results and discussion

Based on the aforementioned established pooling fund model, this section calculates the revenue and expenditure of the UEBMI pooling fund from 2023 to 2050. The forecasting period covers 27 years, qualifying it as a medium-to-long-term forecast. Two schemes were devised in this study to better illustrate the policy effects of OCR: Partial Outpatient Coordination Reform and Complete Outpatient Coordination Reform. Different reimbursement rates for outpatient expenses were applied in each scheme. The baseline scenario, where outpatient coordination reform policies are not implemented, is compared and analyzed against the revenue and expenditure situations under partial and complete outpatient coordination reform schemes.

### 4.1 Fund revenue and expenditure without the implementation of OCR

Based on the aforementioned parameters, this study initially predicts the number of participants in the UEBMI with the cohort–component method. Then, it examines the revenue and expenditure of the pooling fund of the UEBMI under the scenario where outpatient coordination reform is not implemented.

#### 4.1.1 Prediction of the number of urban employees participating in the UEBMI

**Figure 1:**
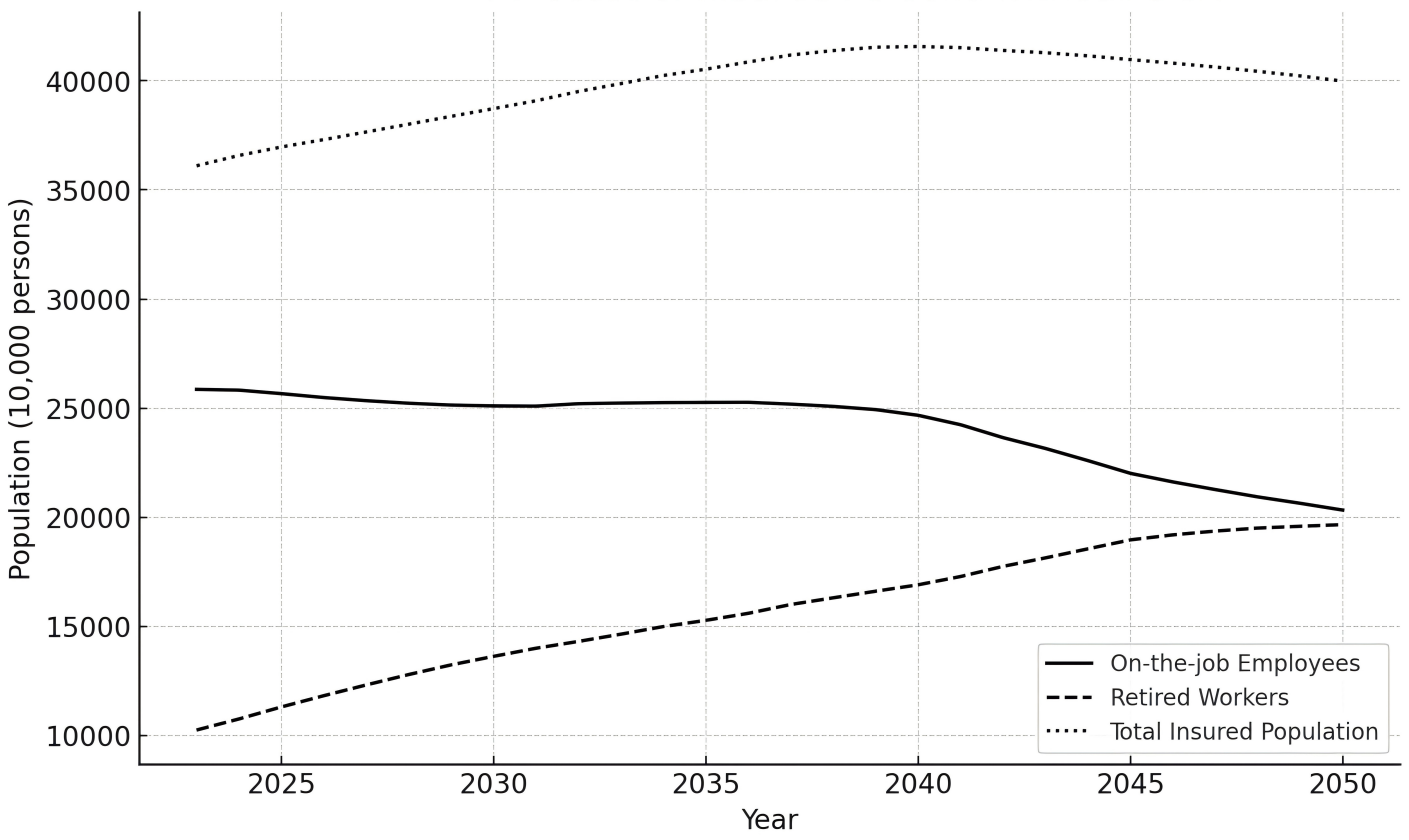
Forecast of insured persons without OCR.

Participants of the UEBMI are divided into two categories: active workers and retirees, depending on whether they are contributing. The specific data and trends in the total number of participants, active workers, and retirees in basic medical insurance for urban employees before 2050 are shown in Table 1 and Figure 1.

**Table 1:** Forecast of insured persons without OCR (unit: 10,000 persons).

**Year**	**On-the-job employees**	**Retired workers**	**Total insured population**
2023	25,861	10,246.09	36,107.09
2024	25,825.44	10,754.88	36,580.32
2025	25,664.46	11,306.03	36,970.48
2026	25,485.8	11,821.52	37,307.33
2027	25,342.03	12,314.82	37,656.86
2028	25,224.25	12,791.18	38,015.43
2029	25,139.5	13,234.37	38,373.87
2030	25,103.43	13,624.12	38,727.55
2031	25,091.88	13,996.71	39,088.59
2032	25,204.04	14,309.58	39,513.62
2033	25,232.71	14,641.49	39,874.2
2034	25,253.42	14,990.93	40,244.34
2035	25,261.8	15,273.81	40,535.6
2036	25,267.65	15,597.04	40,864.69
2037	25,183.29	16,001.17	41,184.46
2038	25,080.45	16,307.17	41,387.63
2039	24,932.06	16,610.15	41,542.22
2040	24,671.28	16,903.31	41,574.58
2041	24,238.55	17,283.19	41,521.74
2042	23,646.87	17,747.56	41,394.42
2043	23,150.67	18,137.61	41,288.28
2044	22,593.15	18,552.5	41,145.65
2045	22,010.87	18,962.23	40,973.1
2046	21,618.42	19,193	40,811.42
2047	21,267.48	19,366.39	40,633.87
2048	20,931.95	19,504.33	40,436.27
2049	20,638.41	19,586.65	40,225.06
2050	20,327.32	19,661.66	39,988.98

Figure 1 shows the change in the number of insured urban employees in China from 2023 to 2050 under the implementation of OCR. The total number of insured urban employees initially rises, peaking at 415.75 million in 2040, before decreasing. Specifically, the number of insured employees gradually decreases from 258.61 million in 2023 to 203.27 million in 2050, with an average annual growth rate of approximately −0.88%. In contrast, the number of insured retired workers increases steadily from 102.46 million in 2023 to 196.62 million in 2050, with an average annual growth rate of about 2.45%. Throughout this period, the number of employees remains higher than the number of retired workers, but the growth rate of the retired population significantly surpasses that of the employees. It is anticipated that, due to population aging, the number of retired workers will continue to exceed the number of active employees in the future.

#### 4.1.2 The revenue and expenditure status of the pooling fund without OCR

**Figure 2:**
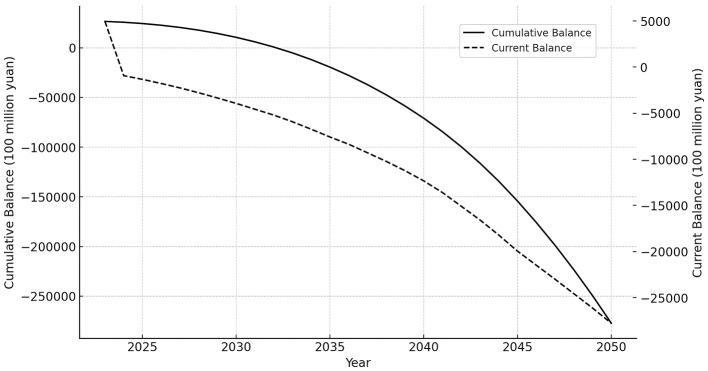
Revenue and expenditure of funds without OCR.

Based on the projected number of insured individuals, the revenue and expenditure status of the UEBMI pooling fund is further simulated by the pooling fund model. The specific trends in the revenue and expenditure of the pooling fund are illustrated in Table 2 and Figure 2.

**Table 2:** Revenue and expenditure of funds without OCR (unit: 100 million yuan).

**Year**	**Fund revenue**	**Fund expenditure**	**Current balance**	**Cumulative balance**
2023	16,260.41	11,298.93	4,961.47	26,610.53
2024	12,504.54	13,426.3	−921.76	25,740.15
2025	13,234.33	14,560.9	−1,326.57	24,462.4
2026	13,930.73	15,702.14	−1,771.41	22,736.37
2027	14,683.27	16,937.12	−2,253.84	20,523.5
2028	15,491.93	18,272.12	−2,780.19	17,778.8
2029	16,366.27	19,711.03	−3,344.76	14,462.91
2030	17,323.36	21,259.51	−3,936.15	10,547.81
2031	18,267.73	22,833.14	−4,565.41	5,994.37
2032	19,358.61	24,558.33	−5,199.73	796.23
2033	20,446.57	26,371.86	−5,925.29	−5,129.07
2034	21,588.83	28,323.63	−6,734.8	−11,863.86
2035	22,783.77	30,362.9	−7,579.12	−19,442.99
2036	23,928.5	32,291.63	−8,363.13	−27,806.11
2037	25,041.04	34,333.61	−9,292.57	−37,098.68
2038	26,185.73	36,407.53	−10,221.81	−47,320.49
2039	27,332.34	38,564.07	−11,231.73	−58,552.21
2040	28,398.77	40,736.84	−12,338.07	−70,890.29
2041	29,156.2	42,761.95	−13,605.76	−84,496.04
2042	29,724.46	44,813.65	−15,089.19	−99,585.23
2043	30,410.27	46,986.42	−16,576.15	−116,161.38
2044	31,013.43	49,224.72	−18,211.29	−134,372.67
2045	31,573.78	51,535.3	−19,961.52	−154,334.19
2046	32,251.25	53,731.22	−21,479.96	−175,814.15
2047	32,996.82	56,001.62	−23,004.81	−198,818.96
2048	33,775.28	58,342.44	−24,567.16	−223,386.12
2049	34,633.7	60,763.18	−26,129.47	−249,515.59
2050	35,476.12	63,249.28	−27,773.15	−277,288.74

Figure 2 describes the trends in the current balance and accumulated balance of the UEBMI pooling fund from 2023 to 2025 under the implementation of OCR. Initially, the current balance shows a trend of gradual decline, decreasing from 496.147 billion yuan in 2023 to −2,777.315 billion yuan in 2050. A current deficit first appears in 2024 and continues to grow. Meanwhile, the accumulated balance experiences a rapid decline, falling from 2,661.053 billion yuan in 2023 to −27,728.874 billion yuan in 2050. In 2033, the accumulated balance goes into deficit and continues to expand. As shown in Table 2, the fund's deficit becomes increasingly severe due to the aging population, as fund expenditures consistently exceed income from 2024 onward. The growth rate of expenditures is higher than that of income, causing the gap between them to widen over time.

### 4.2 The revenue and expenditure of the UEBMI pooling fund with partial OCR

It is supposed that China's OCR will be fully implemented in 2024. Part of the parameter setting consists of the guidance issued by the *General Office of the State Council on establishing and perfecting the basic medical insurance for employee outpatient coordination safeguard mechanism guidance*“ (issued by State Council [2021] No. 14), which allocates the entire employer's share of medical insurance contributions into the pooling fund, rather than to the individual fund. Moreover, the ordinary outpatient expenses are concluded into the pooling fund reimbursement, and individual contribution still belongs to the individual fund. Under the plan of partial OCR, the fund revenue and expenditure with reimbursement ratios of 50 and 75.6% were determined, respectively. The specific situation is shown in Table 3.

**Table 3:** Fund revenue and expenditure of different reimbursement ratios with partial OCR (unit: 100 million yuan).

**Year**	**50% Outpatient expense Reimbursement rate**				**75.6% Outpatient expense Reimbursement rate**			
	**Fund revenue**	**Fund expenditure**	**Current balance**	**Cumulative balance**	**Fund revenue**	**Fund expenditure**	**Current balance**	**Cumulative balance**
2023	16,260.41	11,298.93	4,961.47	26,610.53	16,260.41	11,298.93	4,961.47	26,610.53
2024	17,293.52	14,577.76	2,715.75	29,637.73	17,293.52	15,167.31	2,126.21	29,047.01
2025	18,302.79	15,811.92	2,490.87	32,474.42	18,302.79	16,452.45	1,850.34	31,235.09
2026	19,265.91	17,052.93	2,212.98	35,065.28	19,265.91	17,744.53	1,521.37	33,118.71
2027	20,306.66	18,396	1,910.65	37,383	20,306.66	19,142.95	1,163.7	34,665.6
2028	21,425.01	19,847.99	1,577.02	39,393.08	21,425.01	20,654.84	770.17	35,835.97
2029	22,634.21	21,413.11	1,221.09	41,069.64	22,634.21	22,284.58	349.63	36,598.41
2030	23,957.84	23,097.53	860.31	42403.96	23,957.84	24,038.59	−80.76	36,590.69
2031	25,263.89	24,808.88	455.01	43,347.53	25,263.89	25,820.45	−556.56	36,106.19
2032	26,772.54	26,685.37	87.17	43,933.37	26,772.54	27,774.41	−1,001.88	35,174.53
2033	28,277.17	28,657.83	−380.66	43,639.81	2,8277.17	29,828.24	−1,551.08	33,690.7
2034	29,856.89	30,780.78	−923.89	42,801.36	29,856.89	32,038.84	−2,181.95	31,571.76
2035	31,509.47	32,998.7	−1,489.23	41,394.75	31,509.47	34,348.24	−2,838.76	28,790.47
2036	33,092.6	35,094.98	−2,002.38	39,471.16	33,092.6	36,530.3	−3,437.69	25,403.48
2037	34,631.23	37,314.29	−2,683.06	36,861.67	34,631.23	38,840.4	−4,209.17	21,236.7
2038	36,214.3	3,567.66	−3,353.36	33,575.33	36,214.3	41,185.65	−4,971.35	16,297.88
2039	37,800.04	41,910.46	−4,110.41	29,523.85	37,800.04	43,623.81	−5,823.76	10,495.07
2040	39,274.89	44,270.04	−4,995.14	24,577.76	39,274.89	46,079.03	−6,804.14	3,698.31
2041	40,322.4	46,467.09	−6,144.69	18,469.94	40,322.4	48,364.12	−8,041.72	−4,343.41
2042	41,108.3	48,692.12	−7,583.82	10,907.9	41,108.3	50,677.89	−9,569.59	−13,913
2043	42,056.76	51,048.37	−8,991.61	1,920.12	42,056.76	53,128.08	−11,071.32	−24,984.33
2044	42,890.92	53,475.03	−10,584.11	−8,681.32	42,890.92	55,651.19	−12,760.27	−37,744.6
2045	43,665.86	55,979.41	−12,313.55	−20,994.87	43,665.86	58,254.79	−14,588.93	−52,333.53
2046	44,602.8	58,356.98	−13,754.19	−34,749.06	44,602.8	60,725.38	−16,122.58	−68,456.11
2047	45,633.89	60,814.52	−15,180.63	−49,929.68	45,633.89	63,278.72	−17,644.83	−86,100.94
2048	46,710.49	63,347.46	−16,636.97	−66,566.65	46,710.49	65,910.03	−19,199.54	−105,300.48
2049	47,897.68	65,966.1	−18,068.43	−84,635.08	47,897.68	68,630	−20,732.33	−126,032.81
2050	49,062.72	68,654.43	−19,591.7	−104,226.78	49,062.72	71,421.86	−22,359.14	−148,391.95

As shown in Table 3, first, when the reimbursement ratio of outpatient expenses is 50%, pooling fund expenditures exceed fund revenue starting in 2033, and the gap continues to widen over time. A current deficit is projected to occur in 2023, with the cumulative deficit expected by 2044. In addition, the cumulative deficit of 2033 will reach 10,422.678 billion yuan by 2050. Second, when the reimbursement ratio of outpatient expenses is 75.6%, the fund expenditure is greater than the fund revenue from 2030, and the gap between the two is increasing. In this scenario, a current deficit appears in 2030, and a cumulative deficit arises in 2041, reaching 14,839.195 billion yuan by 2050. As the reimbursement ratio for outpatient expenses increases, both the onset of the current and cumulative deficits occur earlier, and the total cumulative deficit grows larger.

### 4.3 The revenue and expenditure of the UEBMI pooling fund with complete OCR

Given that the impact of partial OCR on the sustainability of the pooling fund is not sufficiently significant, it is proposed to transfer the individual fund into the pooling fund and establish an outpatient coordination security fund. This approach aims to assess the revenue and expenditure status of the UEBMI pooling fund with the complete OCR. The implementation of the complete OCR involves allocating both the employers' and employees' contributions to the pooling fund and eliminating the individual fund, with all inpatient and outpatient expenses being reimbursed by the pooling fund. The financial status of the fund, with reimbursement ratios of 50 and 75.6% under this reform, has been determined, as shown in Table 4.

**Table 4:** Fund revenue and expenditure of different reimbursement rates with complete OCR (unit: 100 million yuan).

**Year**	**50% outpatient expense reimbursement rate**				**75.6% outpatient expense reimbursement rate**			
	**Fund revenue**	**Fund expenditure**	**Current balance**	**Cumulative balance**	**Fund revenue**	**Fund expenditure**	**Current balance**	**Cumulative balance**
2023	16,260.41	11,298.93	4,961.47	26,610.53	16,260.41	11,298.93	4,961.47	26,610.53
2024	22,614.6	14,577.76	8,036.84	34,969.46	22,614.6	15,167.31	7,447.29	34,378.73
2025	23,934.42	15,811.92	8,122.5	43,510.35	23,934.42	16,452.45	7,481.97	42,271.02
2026	25,193.88	17,052.93	8,140.95	52,167.95	25,193.88	17,744.53	7,449.34	50,221.38
2027	26,554.86	18,396	8,158.85	60,943.05	26,554.86	19,142.95	7,411.9	58,225.65
2028	28,017.32	19,847.99	8,169.33	69,829.56	28,017.32	20,654.84	7,362.48	66,272.46
2029	29,598.58	21,413.11	8,185.47	78,834.44	29,598.58	22,284.58	7,314	74,363.22
2030	31,329.48	23,097.53	8,231.95	87,989.45	31,329.48	24,038.59	7,290.89	82,523.86
2031	33,037.39	24,808.88	8,228.52	97,246.3	33,037.39	25,820.45	7,216.94	90,704.26
2032	35,010.24	26,685.37	8,324.87	106,706.16	35,010.24	27,774.41	7,235.83	98,997.66
2033	36,977.83	28,657.83	8,320.01	116,269.92	36,977.83	29,828.24	7,149.59	107,300.02
2034	39,043.63	30,780.78	8,262.85	125,886.4	39,043.63	32,038.84	7,004.78	115,552.76
2035	41,204.7	32,998.7	8,205.99	135,556.5	41,204.7	34,348.24	6,856.46	123,751.79
2036	43,274.94	35,094.98	8,179.96	145,311.72	43,274.94	36,530.3	6,744.65	131,933.07
2037	45,286.99	37,314.29	7,972.7	154,971.45	45,286.99	38,840.4	6,446.59	139,909.79
2038	47,357.17	39,567.66	7,789.5	164,558.7	47,357.17	41,185.65	6,171.52	147,702.61
2039	49,430.83	41,910.46	7,520.37	173,986.54	49,430.83	43,623.81	5,807.02	155,219.83
2040	51,359.48	44,270.04	7,089.44	183,091.01	51,359.48	46,079.03	5,280.45	162,295.86
2041	52,729.29	46,467.09	6,262.2	191,471.28	52,729.29	48,364.12	4,365.17	168,536.16
2042	53,757.01	48,692.12	5,064.89	198,748.22	53,757.01	50,677.89	3,079.12	173,559.6
2043	54,997.3	51,048.37	3,948.94	204,990.66	54,997.3	53,128.08	1,869.22	177428.5
2044	56,088.12	53,475.03	2,613.09	209,966.37	56,088.12	55,651.19	436.93	179,906.73
2045	57,101.51	55,979.41	1,122.1	213,505.33	57,101.51	58,254.79	−1,153.28	179,110.96
2046	58,326.73	58,356.98	−30.25	213,902.02	58,326.73	60,725.38	−2,398.65	177,065.73
2047	59,675.09	60,814.52	−1,139.43	213,188.12	59,675.09	63,278.72	−3,603.63	173,809.03
2048	61,082.95	63,347.46	−2,264.51	211,345.46	61,082.95	65,910.03	−4,827.08	169,319.91
2049	62,635.42	65,966.1	−3,330.68	208,430.81	62,635.42	68,630	−5,994.58	163,651.98
2050	64,158.95	68,654.43	−4,495.48	204,343.2	64,158.95	71,421.86	−7,262.92	156,701.85

As shown in Table 4, when the reimbursement ratio for outpatient expenses is 50%, fund expenditures exceed fund revenue from 2046, and the gap between them continues to widen. A current deficit appears in 2046, and there is no cumulative balance during the forecast period, with the cumulative balance reaching 20,434.32 billion yuan by 2050. Conversely, when the reimbursement ratio for outpatient expenses is 75.6%, fund expenditures surpass fund revenue from 2045 onward, with an increasing gap between the two. A current deficit emerges in 2045, and there is no cumulative balance within the forecast period, with the cumulative balance reaching 15,670.185 billion yuan by 2050. This shows that as the reimbursement rate for outpatient expenses increases, the point at which the current fund balance shows a deficit occurs earlier, and the cumulative balance decreases.

**Figure 3:**
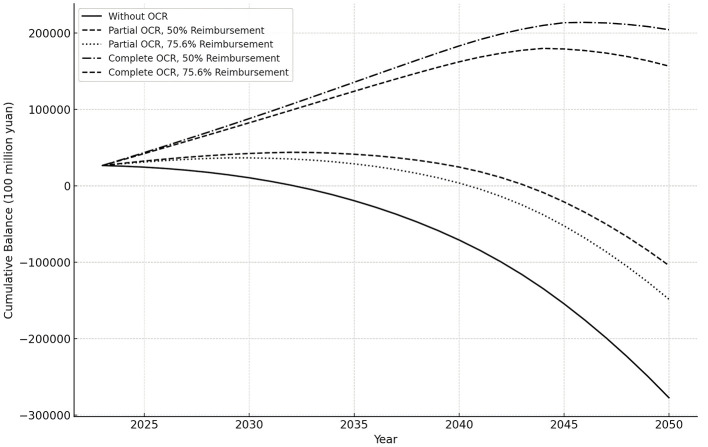
Cumulative balance forecast under various OCR and reimbursement ratios.

Figure 3 shows a comparison chart of the accumulated balance before and after the OCR (including partial and complete) from 2023 to 2050. First, the accumulated balance of the pooling fund after the implementation of the outpatient coordination reform (including partial and complete) is better than that without any policy intervention. Specifically, the point at which the accumulated deficit initially occurs later, and the amount of the accumulated deficit is reduced. Second, regardless of the reimbursement ratio, the accumulated balance of the complete OCR is more than that of the partial OCR, and the time point of the deficit is more backward. Third, with partial OCR, when the outpatient expense reimbursement ratio is 50%, the first cumulative deficit is delayed by 3 years, and the larger the reimbursement ratio, the less the cumulative balance. With the complete OCR, neither reimbursement ratio results in a cumulative deficit during the forecast period. However, as shown in Figure 3, the cumulative balance is higher when the outpatient expense reimbursement ratio is 50% compared to 75.6%.

### 4.4 Robustness test

Due to the large number of parameter settings in this study, to test whether some parameter settings will affect the main conclusions, the stability analysis of the growth rate of contribution earnings and 3-month fixed deposit rate of banks is made according to the study by Yi et al. (6).

#### 4.4.1 Reduce the growth rate of the payment base by 0.5%

As shown in Table 5, holding all other factors constant, the change in the growth rate of the medical insurance fund for urban employees will not affect the main conclusion of this study. First, according to the current system, if OCR is not implemented, the current deficit will appear for the first time in 2024, and the cumulative deficit will appear for the first time in 2033, with the cumulative balance by 2050 reaching 25,686.687 billion yuan. Second, when the growth rate of the contribution base of the UEBMI is reduced by 0.5%, The timing of both the initial occurrence of the current deficit and the accumulated deficit will be delayed. Third, if the OCR is implemented when the reimbursement rate of outpatient expenses is raised from 50 to 75.6%, the role of OCR in improving the revenue and expenditure of the pooling fund will be weakened.

**Table 5:** Robustness analysis of payment base growth rate reduction by 0.5% (unit: 100 million yuan).

**Circumstances**	**No policy implemented**	**Partial OCR**		**Complete OCR**	
		**Reimbursement rate 50%**	**Reimbursement rate 75.6%**	**Reimbursement rate 50%**	**Reimbursement rate 75.6%**
Cumulative balance in 2023	26,610.53	26,610.53	26,610.53	26,610.53	26,610.53
Current deficit period	2024–2050	2032–2050	2030–2050	2046–2050	2044–2050
First year of cumulative deficit	2033–2050	2044–2050	2041–2050	——	——
Accumulated balance in 2050	−256,866.87	−96,415.32	−136,848.19	190,713.62	145,010.01

#### 4.4.2 The 3-month fixed deposit rate of the bank is 2%

As shown in Table 6, holding all the factors constant, the change in bank interest rate will not affect the main conclusion of this study. First, under the current system, if the reform policy of outpatient coordination is not implemented, the current deficit is projected to occur for the first time in 2024, with a cumulative deficit emerging in 2033. By 2050, the cumulative balance is expected to reach 27,728.874 billion yuan. Second, when the 3-month fixed deposit rate of banks is set at 2%, after the implementation of OCR, both the current deficit and the cumulative fund deficits will be delayed for the first time, with the cumulative balance increasing in 2050. Third, if the reimbursement rate of outpatient expenses increases from 50 to 75.6%, the role of the OCR in improving the revenue and expenditure balance of the medical insurance fund is weakened.

**Table 6:** Analysis of robustness of the 3-month fixed deposit rate of the bank by 2% (unit: 100 million yuan).

**Circumstances**	**No policy implemented**	**Partial OCR**		**Complete OCR**	
		**Reimbursement rate 50%**	**Reimbursement rate 75.6%**	**Reimbursement rate 50%**	**Reimbursement rate 75.6%**
Cumulative balance in 2023	26,610.53	26,610.53	26,610.53	26,610.53	26,610.53
Current deficit period	2024–2050	2032–2050	2030–2050	2046–2050	2045–2050
First year of cumulative deficit	2033–2050	2044–2050	2041–2050	——	——
Accumulated balance in 2050	−277,288.74	−101,150.039	−146,657.47	231,023.28	178,970.05

In conclusion, adjusting the main parameters of the model does not significantly affect the main conclusions of this study, so the conclusions are relatively robust.

## 5 Conclusion and policy recommendations

China has initiated the implementation of OCR policies to fully leverage the mutual assistance function of employees' medical insurance. What kind of impact will the implementation of outpatient coordination reform have on the sustainability of medical insurance funds for urban employees? This study focuses on the UEBMI pooling fund as the research subject. By establishing a pooling fund model, it simulates the revenue and expenditure scenarios under different outpatient coordination reform schemes.

The study found:

(1) Without implementing OCR policies, according to the current system, the UEBMI pooling fund will experience deficits in both the current period and cumulative deficits in 2024 and 2033, respectively. By the end of the forecast period in 2050, the cumulative deficit is projected to reach 27,728.874 billion yuan, and the fund's financial situation will deteriorate further.
(2) After the implementation of OCR policies, the financial situation of the pooling fund has improved, and the policy effect of complete OCR is better than that of partial OCR. Under the partial OCR, when the reimbursement rate of outpatient expenses is 50 or 75.6%, the timing of the first cumulative deficit is delayed by 11 and 8 years, respectively, and the cumulative deficit scale is also reduced. Under the complete OCR plan, when the reimbursement ratio of outpatient expenses is 50 or 75.6%, the fund has no cumulative deficit during the forecast period.
(3) Increasing the reimbursement ratio for outpatient expenses weakens OCR's policy effectiveness, causing deficits to occur earlier and reducing the cumulative surplus. Under partial OCR, when the reimbursement rate of outpatient expenses increased from 50 to 75.6%, the point of accumulated deficit occurred 3 years in advance, and the cumulative deficit increased by 4,416.517 billion yuan to 2050. Under the complete OCR, when the reimbursement rate of outpatient expenses increased from 50 to 75.6%, the accumulated balance during the forecast period showed no deficit. Still, the cumulative balance will decrease by 4,764.135 billion yuan by 2050.

While this study provides valuable insights into the sustainability of the pooling fund of China's UEBMI with OCR, it is important to note its primary limitation. The analysis is deeply rooted in China's specific policy context and healthcare system. In addition, the reforms discussed, along with the data used, are unique to China's medical insurance structure, which may limit the applicability of these findings to other countries with different healthcare systems.

Based on the above research conclusions, this study proposes the following policy recommendations:

(1) Promoting the complete OCR policies is recommended as soon as possible, striving to alleviate better the payment pressure on the UEBMI pooling fund. The aforementioned data show that the implementation of the OCR policy can delay the time point of the current deficit and the cumulative deficit of the fund. At present, OCR has not been fully implemented. It is suggested to accelerate the reform process to relieve the pressure of pooling fund payments.

(2) Implementing complete OCR on time is recommended, but the reimbursement ratio needs to be controlled. The empirical results of the above show that the policy effect of complete OCR is better than that of partial OCR. However, with the continuous increase of the reimbursement ratio of outpatient expenses, the solvency of the pooling fund is reduced. Therefore, in the future, to further improve the sustainability and enhance the solvency of the UEBMI pooling fund, the individual fund system should be further reformed. When the time is ripe, the reform can be implemented, but the reimbursement ratio of outpatient expenses should be controlled.

(3) When implementing outpatient coordination reform policies, it is necessary to formulate other supporting policies promptly. In the early stage of the implementation of OCR, all employer contributions will be transferred into the pooling fund, which will increase the fund revenue significantly. Due to the lower average outpatient expense and reimbursement ratio, the fund expenditure will not increase significantly. At this time, the balance of the pooling fund in the current period increases more. However, in the later stages of implementing the OCR policy, as the average outpatient expense per visit and the reimbursement ratio increase, the fund expenditure scale will continue to expand, eventually leading to a cumulative deficit. Therefore, while implementing OCR, it is necessary to introduce additional matching policies to reduce the deficit and ensure the long-term sustainability of the UEBMI pooling fund.

## 6 Outlook

Building upon the findings of this study, future research could delve into more directly related areas to enhance our understanding of OCR's impact on the sustainability of the UEBMI:

Further exploration of outpatient and inpatient synergies: While this study primarily focuses on outpatient coordination, future research could investigate the interaction between outpatient and inpatient cost-sharing structures.

Differentiating effects across employee demographics: This study treats urban employees as a single group, but future research could offer more granularity by exploring the differential impacts of OCR on various demographic groups within urban employees, such as age, income level, and health status.

Simulating policy adjustments based on economic fluctuations and healthcare cost projections: Since OCR is implemented within a dynamic economic environment, future research could model the fund's responsiveness to economic fluctuations, such as periods of economic contraction or rapid healthcare cost inflation.

These research directions aim to directly extend the findings of this study by exploring OCR in various contexts and among different employee demographics. By doing so, future studies can provide nuanced insights to inform more precise policy adjustments, ensuring the fund's sustainability and equitable support for diverse healthcare needs.

## Data Availability

The datasets presented in this study can be found in online repositories. The names of the repository/repositories and accession number(s) can be found below: https://www.stats.gov.cn/sj/pcsj/rkpc/d7c/202303/P020230301403217959330.pdf.
